# Supplementary material for: Advancing Esophageal Cancer Treatment: Immunotherapy in Neoadjuvant and Adjuvant Settings
Source: Cancers (Basel). 2024 Jan 11;16(2):318. doi: 10.3390/cancers16020318 (PMC10813716; doi:10.3390/cancers16020318)
Supplement: Supplementary file 1 [file cancers-16-00318-s001.zip › cancers-2767533-supplementary.pdf]

Supplemental Index

Trials number	Phase	Status	Patient n	EAC/ESCC	Intervention Arms	Primary outcome
NCT04929392 [1]	Phase II	Active, not recruiting	3	EAC or ESCCs	Arm 1: chemoradiation + pembrolizumab + lenvatinib	CR
NCT02998268 [2]	Phase II	Active, not recruiting	39	EACs	Arm 1: conventional induction chemotherapy.<br>Arm 2: pembrolizumab + induction chemotherapy	DFS
NCT03064490 [3]	Phase II	Active, not recruiting	38	EACs or EGAs	Arm 1: chemoradiation + pembrolizumab	Pathologic CR
NCT03544736 [4]	Phase I/II	Active, not recruiting	30	EAC or ESCCs	Arm 1: Nivolumab + radiation<br>Arm 2: Nivolumab + radiation + chemotherapy<br>Arm 3: Nivolumab + chemoradiotherapy + surgery	AEs
NCT03399071 [5]	Phase II	Active, not recruiting	44	EACs or EGAs	Arm 1: FLOT + Avelumab	pCR
NCT03490292 [6]	Phase I/II	Active, not recruiting	22	EAC or ESCCs	Arm 1: Avelumab + Chemoradiation	pCR, DLT
NCT04435197 [7]	Phase II	Recruiting	143	ESCCs	Arm 1: Pembrolizumab + chemotherapy<br>Arm 2: Surgery (Ivor-Lewis or McKeown esophagectomy)	pCR
NCT04813523 [8]	Phase II	Recruiting	30	EGJ adenocarcinoam	Arm 1: Pembrolizumab+Cisplatin+5-FU	MPR
NCT05281003 [9]	Phase II	Recruiting	128	ESCCs	Arm 1: Pembrolizumab + Chemotherapy	pCR, Major hypoxia signals
NCT04389177 [10]	Phase II	Active, not recruiting	50	ESCCs	Arm 1: Pembrolizumab+ Paclitaxel+Cisplatin	MPR
NCT06006650 [11]	Phase II	Recruiting	114	ESCCs	Arm 1: Pembrolizumab + Albumin Paclitaxel and Cisplatin<br>Arm 2: Pembrolizumab + 5-fluorouracil and Cisplatin<br>Arm 3: 5-fluorouracil and Cisplatin	pCR
NCT05541445 [12]	Phase I/II	Recruiting	40	ESCCs	Arm 1: Pembrolizumab + chemoradiotherapy	MPR
NCT04644250 [13]	Phase II	Recruiting	32	ESCCs	Arm 1: Toripalimab with chemoradiotherapy	pCR
NCT05174325 [14]	Phase II	Recruiting	30	ESCCs	Arm 1: Sintilimab + chemotherapy	pCR
NCT05491616 [15]	Phase II	Recruiting	74	EAC or ESCCs	Arm 1: Nivolumab	DFS
NCT04229459 [16]	Phase II	Recruiting	31	ESCCs	Arm 1: chemoradiation + cetuximab + nivolumab	pCR, PFS, adverse events
NCT05213312 [17]	Phase II/III	Recruiting	90	ESCCs	Arm 1: Nivolumab + chemotherapy + Surgery + Nivolumab<br>Arm 2: Chemotherapy + Surgery + Nivolumab	pCR
NCT05130684 [18]	Phase II	Recruiting	43	ESCCs	Arm 1: Nivolumab + Paclitaxel + Cisplatin + RT	Treatment related death, completion of treatment
NCT04804696 [19]	Phase II	Recruiting	53	ESCCs	Arm 1: Toripalimab + Paclitaxel + Cisplatin	pCR
NCT04159974 [20]	Phase II	Recruiting	56	EACs	Arm 1: Durvalumab<br>Arm 2: Durvalumab + Tremelimumab	Safety and efficacy
NCT04221555 [21]	Phase II	Recruiting	68	EGJ and GACs	Arm 1: Docetaxel, Oxaliplatin, S-1 and Durvalumab + surgery + Durvalumab<br>Arm 2: Durvalumab and Tremelimumab + surgery + Durvalumab	pCR
NCT04568200 [22]	Phase II	Recruiting	60	ESCCs	Arm 1: Durvalumab + chemoradiotherapy<br>Arm 2: Chemoradiotherapy	tumor response and pathological response
NCT03784326 [23]	Phase II	Recruiting	40	EACs	Arm 1: Atezolizumab + chemotherapy + Surgery + Atezolizumab<br>Arm 2: Atezolizumab + Tiragolumab + chemotherapy + Surgery + Atezolizumab + Tiragolumab	pCR
NCT05836584 [24]	Phase II	Recruiting	240	EGJ and GACs	Arm 1: Chemotherapy + Atezolizumab + surgery + Chemotherapy and atezolizumab<br>Arm 2: Atezolizumab + Surgery + Atezolizumab	EFS

NCT05817201 [25]	Phase II/III	Recruiting	60	EAC or ESCCs	Arm 1: Toripalimab + Radiotherapy<br>Arm 2: Chemotherapy + Radiotherapy	OS
NCT05424432 [26]	Phase II	Recruiting	63	ESCCs	Arm 1: chemoradiotherapy + toripalimab	pCR
NCT04280822 [27]	Phase II	Recruiting	400	ESCCs	Arm 1: Toripalimab + chemotherapy + surgery + Toripalimab<br>Arm 2: Chemotherapy + surgery	EFS
NCT05994456 [28]	Phase II	Recruiting	24	EGJ and GACs	Arm 1: Toripalimab	pCR
NCT05777707 [29]	Phase I/II	Recruiting	89	EAC or ESCCs	Arm 1: Sintilimab/Camrelizumab/Toripalimab/Tislelizumab + chemotherapy	DFS
NCT04177797 [30]	Phase II	Active, not recruiting	20	ESCCs	Arm 1: Toripalimb	pCR
NCT04212598 [31]	Phase II	Recruiting	40	ESCCs	Arm 1: Chemoradiotherapy + Sintilimab	percentage of disease progression
NCT05357846 [32]	Phase III	Active, not recruiting	422	ESCCs	Arm 1: Sintilimab + Chemoradiotherapy + Surgery<br>Arm 2: Chemoradiotherapy + Surgery	OS

Table S1: Neoadjuvant immunotherapy ongoing clinical trials of ESCCs and EACs. GEJ**:** gastroesophageal junction; ESCC: esophageal squamous cell carcinoma; GAC: gastric adenocarcinoma.

**Immunotherapy**	**Trial Number**	**Phase**	**Status**	**Patients** n	**Inclusion Criteria**	**Intervention**	**Primary Endpoints**
Pembrolizumab	NCT04210115 (KEYNOTE-975) [33]	Phase III	Recruiting	700	CTX N+ M0 or cT2-T4a NX M0 ESCC, GEJC, EAC	Pembrolizumab+FP	EFS, OS
	NCT04807673 (KEYSTONE-002) [34]	Phase III	Recruiting	342	ESCC	Pembrolizumab + Chemotherapy	EFS
Atezolizumab	NCT04543617 (SKYSCRAPER-07) [35]	Phase III	Recruiting	750	ESCC	Tiragolumab + Atezolizumab	OS, PFS
Durvalumab	NCT04550260 (KUNLUN) [36]	Phase III	Recruiting	600	ESCC	Durvalumab + definitive CRT	PFS
Toripalimab	NCT04848753 [37]	Phase III	Active, not recruiting	663	ESCC	Toripalimab + cisplatin and paclitaxel	EFS

Table S2: Ongoing phase III clinical trials of immunotherapy combined with chemotherapy/chemoradiotherapy in ESCCs and EACs

1. City of Hope Medical Center. A Phase 2 Trial of Neoadjuvant Chemoradiation With Pembrolizumab Followed by Pembrolizumab With Lenvatinib in Esophageal/Gastroesophageal Junction Squamous Cell and Adenocarcinomas [Internet]. clinicaltrials.gov; 2023 [cited 2023 Dec 31]. Available from: https://clinicaltrials.gov/study/NCT04929392

2. Weill Medical College of Cornell University. Randomized, Multicenter Phase II Study of Pembrolizumab in Combination With Chemotherapy and Chemoradiation in Locally Advanced Esophageal Adenocarcinoma [Internet]. clinicaltrials.gov; 2023 [cited 2023 Dec 31]. Available from: https://clinicaltrials.gov/study/NCT02998268

3. Duke University. Pembrolizumab, Radiotherapy, and Chemotherapy in Neoadjuvant Treatment of Malignant Esophago-gastric Diseases (PROCEED) [Internet]. clinicaltrials.gov; 2023 [cited 2023 Dec 31]. Available from: https://clinicaltrials.gov/study/NCT03064490

4. Hjortland GO. Safety and Feasibility of Irradiation and Nivolumab in Esophageal Cancer (INEC-study) - a Phase I/II Trial [Internet]. clinicaltrials.gov; 2023 [cited 2023 Dec 31]. Available from: https://clinicaltrials.gov/study/NCT03544736

5. Royal Marsden NHS Foundation Trust. Study Title: Peri-operative Immuno-Chemotherapy in Operable Oesophageal and Gastric Cancer (ICONIC Trial) [Internet]. clinicaltrials.gov; 2023 [cited 2023 Dec 31]. Available from: https://clinicaltrials.gov/study/NCT03399071

6. University of Wisconsin, Madison. Phase I/II Trial of Avelumab in Combination With Chemoradiation in the Treatment of Stage II/III Resectable Esophageal and Gastroesophageal Cancer [Internet]. clinicaltrials.gov; 2022 [cited 2023 Dec 31]. Available from: https://clinicaltrials.gov/study/NCT03490292

7. M.D HL. Multicenter Preoperative Anti-PD-1 Antibody Combined With Chemoradiotherapy for Locally Advanced Squamous Cell Carcinoma of Esophageus [Internet]. clinicaltrials.gov; 2023 [cited 2023 Dec 31]. Available from: https://clinicaltrials.gov/study/NCT04435197

8. Tianjin Medical University Cancer Institute and Hospital. Efficacy and Safety of Pembrolizumab Plus Neoadjuvant Chemotherapy With Cisplatin and 5-Fluorouracill Followed by Surgery in Patients With Locally Advanced Adenocarcinoma of Esophagogastric Junction [Internet]. clinicaltrials.gov; 2021 [cited 2023 Dec 31]. Available from: https://clinicaltrials.gov/study/NCT04813523

9. Chen H. A Pilot Study of Hypoxia as a Potential Resistance Mechanism to PD-1 Checkpoint Blockade Therapy in Neoadjuvant Treatment of Esophageal Squamous Cell Carcinoma (HYPERION) [Internet]. clinicaltrials.gov; 2023 [cited 2023 Dec 31]. Available from: https://clinicaltrials.gov/study/NCT05281003

10. Tianjin Medical University Cancer Institute and Hospital. Efficacy and Safety of Pembrolizumab Plus Paclitaxel, Cisplatin Followed by Surgery in Patients With Locally Advanced Esophageal Squamous Cell Carcinoma (KEYSTONE-001) [Internet]. clinicaltrials.gov; 2023 [cited 2023 Dec 31]. Available from: https://clinicaltrials.gov/study/NCT04389177

11. yanxiaolong. Pembrolizumab Plus Albumin Paclitaxel or 5-fluorouracil and Cisplatin Versus 5-fluorouracil and Cisplatin in Neoadjuvant Therapy for Resectable Esophageal Squamous Cell Carcinoma: a Prospective, Randomized Controlled Trial Study [Internet]. clinicaltrials.gov; 2023 [cited 2023 Dec 31]. Available from: https://clinicaltrials.gov/study/NCT06006650

12. Cancer Institute and Hospital, Chinese Academy of Medical Sciences. A Prospective, Single-arm Phase Ib/II Study to Explore the Safety and Efficacy of Pembrolizumab Combined With Neoadjuvant Chemoradiotherapy (CRT) Followed by Surgery for Upper Locally Advanced Esophageal Squamous Cell Carcinoma (ESCC)

[Internet]. clinicaltrials.gov; 2022 [cited 2023 Dec 31]. Available from: https://clinicaltrials.gov/study/NCT05541445

13. Ren W. Phase II of Toripalimab Chemoradiotherapy in Neoadjuvant Treatment of Locally Advanced Esophageal Squamous Cell Carcinoma: A Single-center、Open Lable、Single-arm Exploratory Clinical Research [Internet]. clinicaltrials.gov; 2020 [cited 2023 Dec 31]. Available from: https://clinicaltrials.gov/study/NCT04644250

14. The First Affiliated Hospital of Soochow University. Phase II Exploratory Study of the Effectiveness of Neoadjuvant Chemotherapy Combined With PD-1 Monoclonal Antibody in the Treatment of Esophageal Squamous Cell Carcinoma: a Prospective, Single-center, Single-arm Study [Internet]. clinicaltrials.gov; 2021 [cited 2023 Dec 31]. Available from: https://clinicaltrials.gov/study/NCT05174325

15. Mostert DB (Bianca). Nivolumab During Active Surveillance After Neoadjuvant Chemoradiation for Esophageal Cancer: SANO-3 Study [Internet]. clinicaltrials.gov; 2022 [cited 2023 Dec 31]. Available from: https://clinicaltrials.gov/study/NCT05491616

16. Brenner B. A Phase II Study of the Addition of Nivolumab and Cetuximab to Chemoradiation in Locally Advanced Esophageal Squamous Cell Carcinoma (ESqCC). [Internet]. clinicaltrials.gov; 2020 [cited 2023 Dec 31]. Available from: https://clinicaltrials.gov/study/NCT04229459

17. Shanghai Zhongshan Hospital. A Randomized, Multicenter, Double Blind, Phase II Study of Neoadjuvant Nivolumab or Placebo Plus Chemotherapy Followed by Surgery and Adjuvant Treatment in Subjects With Resectable Esophageal Squamous Cell Carcinoma [Internet]. clinicaltrials.gov; 2022 [cited 2023 Dec 31]. Available from: https://clinicaltrials.gov/study/NCT05213312

18. National Taiwan University Hospital. Neoadjuvant Nivolumab Plus Paclitaxel/ Cisplatin- Chemo-Radiotherapy (Neo-NTP-CRT) Followed by Esophagectomy for Locally Advanced Esophageal Squamous Cell Carcinoma (ESCC) [Internet]. clinicaltrials.gov; 2021 [cited 2023 Dec 31]. Available from: https://clinicaltrials.gov/study/NCT05130684

19. Zhang X. Toripalimab With Paclitaxel and Cisplatin as Neoadjuvant Treatment for Esophageal Squamous Cell Carcinoma [Internet]. clinicaltrials.gov; 2023 [cited 2023 Dec 31]. Available from: https://clinicaltrials.gov/study/NCT04804696

20. Zander T. A Phase II Trial to Evaluate Safety and Efficacy of Adding Durvalumab (MEDI4736) to Standard Neoadjuvant Radiochemotherapy and of Adjuvant Durvalumab +/- Tremelimumab in Locally Advanced Esophageal Adenocarcinoma and to Evaluate Biomarkers Predictive for Response to Immune Checkpoint Inhibition [Internet]. clinicaltrials.gov; 2022 [cited 2023 Dec 31]. Available from: https://clinicaltrials.gov/study/NCT04159974

21. Ryu M-H. Neoadjuvant Durvalumab (MEDI4736) Plus Docetaxel, Oxaliplatin, S-1 (DOS) Followed by Surgery and Adjuvant Durvalumab Plus S-1 Chemotherapy in Potentially Resectable MMR Proficient (pMMR) Gastric or Gastroesophageal Junction (GEJ) Adenocarcinoma [Internet]. clinicaltrials.gov; 2023 [cited 2023 Dec 31]. Available from: https://clinicaltrials.gov/study/NCT04221555

22. Peking Union Medical College Hospital. A Prospective, Randomized Controlled Study to Evaluate the Efficacy and Safety of Durvalumab Combined With Neoadjuvant Therapy in Patients With Local Advanced Esophageal Squamous Cell Carcinoma [Internet]. clinicaltrials.gov; 2022 [cited 2023 Dec 31]. Available from: https://clinicaltrials.gov/study/NCT04568200

23. M.D. Anderson Cancer Center. Phase I/II Study of Perioperative Chemotherapy Plus Immunotherapy Followed by Surgery in Localized Esophageal and Gastroesophageal Adenocarcinoma [Internet]. clinicaltrials.gov; 2023 [cited 2023 Dec 31]. Available from: https://clinicaltrials.gov/study/NCT03784326

24. National Cancer Institute (NCI). A Randomized Phase II Study of Perioperative Atezolizumab +/- Chemotherapy in Resectable MSI-H/dMMR Gastric and Gastroesophageal Junction (GEJ) Cancer [Internet]. clinicaltrials.gov; 2023 [cited 2023 Dec 31]. Available from: https://clinicaltrials.gov/study/NCT05836584

25. Yang H. A Phase II and III, Randomized, Multicenter Clinical Study: Toripalimab Plus Radiotherapy for Elderly Esophageal Cancer Patients Treated With Non-chemotherapy Strategy [Internet]. clinicaltrials.gov; 2023 [cited 2023 Dec 31]. Available from: https://clinicaltrials.gov/study/NCT05817201

26. Jiang N. Short Course Neoadjuvant Chemo-radiotherapy Plus Anti-PD-1 Antibody (Toripalimab) for Locally Advanced Resectble Squamous Cell Carcinoma of Esophagus (SCALE-2） [Internet]. clinicaltrials.gov; 2022 [cited 2023 Dec 31]. Available from: https://clinicaltrials.gov/study/NCT05424432

27. Henan Cancer Hospital. A Phase III, Randomized Controlled Study of Neo-adjuvant Toripalimab (JS001) in Combination With Chemotherapy Versus Neo-adjuvant Chemotherapy for Resectable Esophageal Squamous Cell Carcinoma [Internet]. clinicaltrials.gov; 2022 [cited 2023 Dec 31]. Available from: https://clinicaltrials.gov/study/NCT04280822

28. Zhang D sheng. Neoadjuvant Toripalimab in the Treatment of Locally Advanced dMMR/MSI-H Gastric or Gastroesophageal Junction Adenocarcinoma：an Open-label, Single-arm， Multi-center， Phase II Trial [Internet]. clinicaltrials.gov; 2023 [cited 2023 Dec 31]. Available from: https://clinicaltrials.gov/study/NCT05994456

29. Beijing Friendship Hospital. Neoadjuvant Therapy of PD-1 Blockade Combined With Chemotherapy for Locally Advanced Esophageal Carcinoma [Internet]. clinicaltrials.gov; 2023 [cited 2023 Dec 31]. Available from: https://clinicaltrials.gov/study/NCT05777707

30. Han Y. A Single Arm Trial of Toripalimab With Neoadjuvant Carboplatin and Paclitaxel for Locally Advanced Esophageal Squamous Cell Carcinoma [Internet]. clinicaltrials.gov; 2022 [cited 2023 Dec 31]. Available from: https://clinicaltrials.gov/study/NCT04177797

31. Xie,MD,PhD C. The Value of Sintilimab Consolidation Therapy After Definitive Concurrent Chemoradiotherapy for Locally Advanced Thoracic Esophageal Squamous Cell Carcinoma, an Open, Prospective, Single-arm Phase II Study [Internet]. clinicaltrials.gov; 2023 [cited 2023 Dec 31]. Available from: https://clinicaltrials.gov/study/NCT04212598

32. Hong Y. Phase III Multicenter Randomized Controlled Trial of PD-1 Inhibitor Combined With Preoperative Concurrent Chemoradiotherapy and Surgery for Locally Advanced Esophageal Squamous Cell Carcinoma [Internet]. clinicaltrials.gov; 2022 [cited 2023 Dec 31]. Available from: https://clinicaltrials.gov/study/NCT05357846

33. Merck Sharp & Dohme LLC. A Randomized, Double-blind, Placebo-controlled Phase 3 Trial of Pembrolizumab (MK-3475) Versus Placebo in Participants With Esophageal Carcinoma Receiving Concurrent Definitive Chemoradiotherapy (KEYNOTE 975) [Internet]. clinicaltrials.gov; 2023 [cited 2023 Dec 31]. Available from: https://clinicaltrials.gov/study/NCT04210115

34. Tianjin Medical University Cancer Institute and Hospital. A Multicenter Randomized, Controlled Phase III Clinical Trial of Pembrolizumab Plus Paclitaxel and Cisplatin Versus Neoadjuvant Chemoradiotherapy Followed by Surgery for Locally Advanced Esophageal Squamous Cell Carcinoma (KEYSTONE-002)

[Internet]. clinicaltrials.gov; 2022 [cited 2023 Dec 31]. Available from: https://clinicaltrials.gov/study/NCT04807673

35. Hoffmann-La Roche. A Phase III, Randomized, Double-Blind, Placebo-Controlled Study of Atezolizumab With or Without Tiragolumab (Anti-TIGIT Antibody) in Patients With Unresectable Esophageal Squamous Cell Carcinoma Whose Cancers Have Not Progressed Following Definitive Concurrent Chemoradiotherapy [Internet]. clinicaltrials.gov; 2023 [cited 2023 Dec 31]. Available from: https://clinicaltrials.gov/study/NCT04543617

36. AstraZeneca. A Phase III, Randomized, Double-Blind, Placebo Controlled, Multi-Center, International Study of Durvalumab Given Concurrently With Definitive Chemoradiation Therapy in Patients With Locally Advanced, Unresectable Esophageal Squamous Cell Carcinoma (KUNLUN) [Internet]. clinicaltrials.gov; 2023 [cited 2023 Dec 31]. Available from: https://clinicaltrials.gov/study/NCT04550260

37. Shanghai Junshi Bioscience Co., Ltd. A Phase III Multicenter, Randomized, Double-blind, Placebo-controlled Study to Evaluate Perioperative Toripalimab (JS001) Combined With Neoadjuvant Chemotherapy in Patients With Resectable Locally Advanced Thoracic Esophageal Squamous Cell Carcinoma [Internet]. clinicaltrials.gov; 2023 [cited 2023 Dec 31]. Available from: https://clinicaltrials.gov/study/NCT04848753
